# Reproductive Effects of Two Neonicotinoid Insecticides on Mouse Sperm Function and Early Embryonic Development *In Vitro*

**DOI:** 10.1371/journal.pone.0070112

**Published:** 2013-07-29

**Authors:** Yi-hua Gu, Yan Li, Xue-feng Huang, Ju-fen Zheng, Jun Yang, Hua Diao, Yao Yuan, Yan Xu, Miao Liu, Hui-juan Shi, Wen-ping Xu

**Affiliations:** 1 Obstetrics & Gynecology Hospital, Institute of Reproduction & Development, Fudan University, Shanghai, P. R. of China; 2 Shanghai Institute of Planned Parenthood Research, Institute of Reproduction & Development, Fudan University, Shanghai, P. R. of China; 3 Reproductive Medical Center, the First Affiliated Hospital of Wenzhou Medical University, Wenzhou, Zhejiang, P. R. of China; 4 School of Pharmacy, East China University of Science and Technology, Shanghai, P. R. of China; State Key Laboratory of Reproductive Biology, Institute of Zoology, Chinese Academy of Sciences, China

## Abstract

Acetamiprid (ACE) and imidacloprid (IMI) are two major members in the family of neonicotinoid pesticides, which are synthesized with a higher selectivity to insects. The present study determined and compared *in vitro* effects of ACE, IMI and nicotine on mammalian reproduction by using an integrated testing strategy for reproductive toxicology, which covered sperm quality, sperm penetration into oocytes and preimplantation embryonic development. Direct chemical exposure (500 µM or 5 mM) on spermatozoa during capacitation was performed, and *in vitro* fertilization (IVF) process, zygotes and 2-cell embryos were respectively incubated with chemical-supplemented medium until blastocyst formation to evaluate the reproductive toxicity of these chemicals and monitor the stages mainly affected. Generally, treatment of 500 µM or 5 mM chemicals for 30 min did not change sperm motility and DNA integrity significantly but the fertilization ability in *in vitro* fertilization (IVF) process**,** indicating that IVF process could detect and distinguish subtle effect of spermatozoa exposed to different chemicals. Culture experiment in the presence of chemicals in medium showed that fertilization process and zygotes are adversely affected by direct exposure of chemicals (*P*<0.05), in an order of nicotine>IMI>ACE, whereas developmental progression of 2-cell stage embryos was similar to controls (*P*>0.05). These findings unveiled the hazardous effects of neonicotinoid pesticides exposure on mammalian sperm fertilization ability as well as embryonic development, raising the concerns that neonicotinoid pesticides may pose reproductive risks on human reproductive health, especially in professional populations.

## Introduction

Pesticides are widespread chemicals mainly utilized in pest control. Only for agricultural purposes, more than 140, 000 tones of pesticides are used annually in the European Union [1], this kind of extensive application raised concerns about hazards of pesticides to human health, including reproductive safety. Imidacloprid (IMI) and acetamiprid (ACE) are two of main compounds in a relatively new class of pesticides, neonicotinoids, which occupied dominant position in global market due to broad spectrum of activity to pests, low residue in environment and low toxicity to human [2], [3]. Despite that they are synthesized to selectively bind to insect nicotinic acetylcholine receptor (nAChRs) in central nervous systems [4], *in vivo* studies demonstrated that IMI, ACE and other neonicotinoid pesticides could adversely affect mammalian reproductive organs such as retardation of testicular development, damage to spermatogenesis, decrease in sperm quality and change of ovary morphology [5], [6], [7], [8], [9].

Sharing partial structure and similar action with nicotine, deleterious effects of IMI and ACE in animal experiments are similar to those of nicotine [10], [11], [12]. To illustrate *in vitro* effects on reproduction and the underlying mechanism, reproductive toxicity study of nicotine exposure on mammalian spermatozoa or oocytes has been conducted for years [13], [14], [15], [16]. However, studies are rarely reported for the *in vitro* effects of IMI and ACE on mammalian reproduction, especially on their direct actions upon fertilization and preimplantation embryonic development. Referring to previous studies of *in vitro* effects of nicotine, we evaluated and compared the impacts of IMI, ACE and nicotine on mouse reproduction by using an integrated testing strategy for reproductive toxicology. To study their direct action upon sperm, sperm parameters were determined, such as motility and DNA integrity. Fertilization capability of the chemical-treated spermatozoa was also estimated by using *in vitro* fertilization (IVF) procedure, which is regarded as a sensitive screening system for reproductive toxicants [17]. The resultant zygotes were kept in culture until blastocyst formation, which could also help determine the impacts of chemical treatment on sperm functions. To evaluate the sensitivities of fertilization and embryos at different preimplantation stages to these chemicals, normal IVF procedure, zygotes and 2-cell embryos were respectively treated with chemicals, cultured in presence of chemicals, and monitored continuously until blastocyst formation.

## Materials and Methods

### Ethical Statement

This study was carried out in full compliance with Guide for the Care and Use of Laboratory Animals. The protocol was approved by the Committee on the Ethics of Animal Experiments of Shanghai Institute of Planned Parenthood Research.

### Chemicals

Nicotine ((−)-nicotine) was purchased from Sigma-Aldrich (St Louis, MO) at purities >99%. The neonicotinoid insecticides 1-(6-chloro-3-pyridylmethyl)-N-nitroimidazolidin-2-ylideneamine (IMI, purity >96%) and (E)-N^1^-[(6-chloro-3-pyridyl) methyl]-N^2^- cyano-N^1^-methylacetamidine (ACE, purity >96%) were acquired from Shanghai Pesticide Research Institute (Shanghai, China). They were dissolved in dimethyl sulfoxide (DMSO, Sigma-Aldrich), and their stock solutions (500 mM) were frozen at −20°C immediately prior to use to minimize their inactivation and degradation.

### Animals

6–8 weeks old female B6D2F1 (C57BL/6×DBA/2) strain mice were used as oocyte donors, and 10–15 weeks old male B6D2F1 mice were used as semen donors. All mice were housed under controlled light conditions (12 h light: 12 h dark) in the Laboratory Animal Services Facility and were fed a standard mouse diet and water *ad libitum*.

### Experimental Design

To investigate the effect of these three substances on fertilization and embryonic development, concentrated ACE, IMI and nicotine were prepared as medium supplements. DMSO was used at final concentration ≤0.1% and this vehicle was used as control to investigate the potential effect of the solvent.

In a previous report, concentrations of 0.1mM to 10 mM were used to approximately represent different ranges of residual nicotine in the testis of light to habitual smokers, wherein the motility of human spermatozoa does not change after exposure to 500 µM of nicotine for 30 minutes [14]. In our preliminary experiment, we also found that nicotine exposure with a dosage of 500 µM for 30 minutes could not impair both motility and fertilization capability of mouse spermatozoa. We then adopted concentration of 5 mM in the sperm exposure experiment.

In sperm exposure experiment, mouse spermatozoa were placed in ACE, IMI or nicotine-containing (500 µM or 5 mM) HTF medium supplemented with bovine serum albumin (HTF-BSA, Sigma) for 30 min first, then washed by and incubated in fresh HTF-BSA medium for another 60 min until capacitation finished, followed by normal IVF procedure. Control spermatozoa were processed with the same procedure except the exposure of chemicals.

To study their effects on the development of early embryos that skipped the stage of fertilization or the first cleavage, zygotes with 2 pronuclei as well as 2-cell stage embryos by natural insemination were cultured in ACE, IMI or nicotine-added KSOM medium (500 µM) to observe how chemicals worked at subsequent developmental stage. Furthermore, the consecutive exposure process from fertilization to blastocyst formation was monitored with exposure concentration of 500 µM both in HTF medium for fertilization and KSOM medium for embryo culture. Concentrations of pesticides were limited to 500 µM, because our preliminary experiments indicated that oocytes and embryos with higher than 500 µM of nicotine would induce massive fragmentation or death the next day.

### Collection of Spermatozoa

Caudal epididymides were isolated, gently squeezed out and placed in a 2.0 ml eppendorf tube with HTF-BSA. “Swim-up” spermatozoa were obtained after incubation at 37°C for 10 min.

### Collection of Oocytes and Embryos

Mature female mice were superovulated with 10 IU of pregnant mare serum gonadotropin (PMSG) and 5 IU of human chorionic gonadotropin (HCG) at 48 h intervals. 14–16 h after HCG administration, cumulus oocyte complexes (COCs) were collected from the removed oviducts and then maintained in human tubal fluid (HTF, Sage In-Vitro Fertilization, Trumbull, CT) medium supplemented with 10% human serum albumin (HSA, Vitrolife, Gothenburg, SE) at 37°C in an atmosphere of 5% CO_2_ in air until use.

With regard to the recovery of the naturally fertilized zygotes and embryos, several female mice were mated with males and examined 12–18 h after HCG injection for the presence of copulation plugs. Fertilized oocytes and 2-cell embryos were recovered by flushing the oviducts 24 h and 40 h later after the HCG injection, respectively. The cumulus of oocytes were dispersed with 0.1% hyaluronidase (Sigma-Aldrich, St. Louis, MO) and washed in several changes of HCZB medium. Fertilized oocytes (identified by the presence of a second polar body and two pronuclei) and 2-cell embryos were then placed in potassium chloride supplemented simplex optimized medium (KSOM medium, Millipore-Chemicon, Billerica, MA), which was designed for culture of implantation stage embryos and previously equilibrated in a humidified atmosphere of 5% CO_2_ in air at 37°C.

### Sperm Motility Assay

The control droplet consisted of an equivalent volume of DMSO in treated groups. After incubation, a 15-µl aliquot of the treated and control samples was transferred into each of two compartments on a glass cannula slide for computer-assisted sperm analysis (CASA) using the integrated visual optical system (IVOS) motility analyzer (Hamilton-Thorne Research, Inc.). Thirty frames were acquired at a frame rate of 60 Hz. The operational settings of the IVOS were as follows: minimum contrast (40) and size (four pixels), gate thresholds 0.38/1.65 for intensity and 0.42/2.34 for size, static elongation 0/75, progressive minimum path velocities of sperm (VAP) 50 µm/sec, straightness threshold 50%, and magnification 0.82.

### Sperm Chromatin Dispersion (SCD) Assay

Generally, SCD assay was developed as the Halosperm® kit (INDAS Laboratories, Madrid, Spain) instructed. An aliquot of each semen sample was diluted to 5–10 million/ml in PBS. The unfixed suspensions were mixed with 1% low-melting-point aqueous agarose (to obtain a 0.7% final agarose concentration) at 37°C. Aliquots of 20 µl mixture were pipetted onto a glass slide precoated with 0.65% standard agarose, covered with a coverslip (22 × 22 mm), and left to solidify at 4°C for 5 min. Then coverslips were carefully removed and slides immediately incubated with freshly prepared acid denaturation solution for 7 min (RT) in the dark to generate restricted single-stranded DNA (ssDNA) motifs from DNA breaks. The denaturation was then stopped, followed by incubation with lysing solution for 23 min (RT). Slides were thoroughly washed in deionized water for 5 min, dehydrated in sequential 70%, 90%, and 100% ethanol baths (2 min each) and air dried. Afterwards, cells were stained with modified Wright-Giemsa stain (Sigma-Aldrich, St. Louis, MO) for bright-field microscopy and a minimum of 400 spermatozoa per sample were evaluated under the × 40 objective of the light microscope. After staining, four SCD patterns were established: sperm heads with (i) large size halos, whose halo width was similar or larger than the minor diameter of the core, (ii) medium size halos, whose halo size was between those with large and with small halo, (iii) small size halos, whose halo width was similar or smaller than one third of the minor diameter of the core, and (iv) without a halo or degraded sperm cells, the latter ones were weakly or irregularly stained. The spermatozoa without DNA damage showed nucleoids with large- or medium-sized halos of spreading DNA loops whereas those with fragmented DNA appeared with a small or no halo. Finally, the percentage of sperm (iii) and (iv) was considered as DNA fragmentation index (DFI) for each semen sample. In this study, spermatozoa pre-incubated in ACE, IMI or nicotine-added HTF medium (5 mM or 500 µM) for 30 min were analyzed for DNA integrity.

### *In vitro* Fertilization and Preimplantation Embryonic Development Procedure

IVF procedure was performed as previously described [18]. HTF medium was equilibrated in a 37°C, 5% CO_2_ incubator one day before experiment. Next day, caudal epididymides were collected from adult male mice. A dense sperm mass was squeezed out and then incubated in HTF-BSA medium for 60–90 min at 37°C to develop their fertilization potential (capacitation). A small volume of capacitated sperm suspension was added to a drop of 200 µl HTF-BSA medium containing freshly ovulated oocytes to achieve a final sperm concentration of 10^6^/ml. Four to six hours later, fertilized oocytes at pronuclear stage were washed and cultured in KSOM for *in vitro* development to morula/blastocyst stages in 5% CO_2_ in air. Oocytes were observed for male and female pronucleus formation (fertilization) at 6 h after the initiation of culture, and the number of 2-cell embryos, 4-cell embryos, morulae and blastocysts after 24 h, 48 h, 72 h and 96 h in culture were checked and recorded respectively.

### Statistical Analysis

SPSS for Windows (Version 15.0; SPSS Inc., Chicago, IL, USA) was used for statistical analysis. *In vitro* developmental outcomes and SCD results were evaluated using *χ^2^* tests and one-way analysis of variance (ANOVA) for significance, respectively. Results were considered statistically significant at *P*<0.05.

## Results

### Inﬂuences of Chemical Exposure on Sperm Function

**Figure 1:**
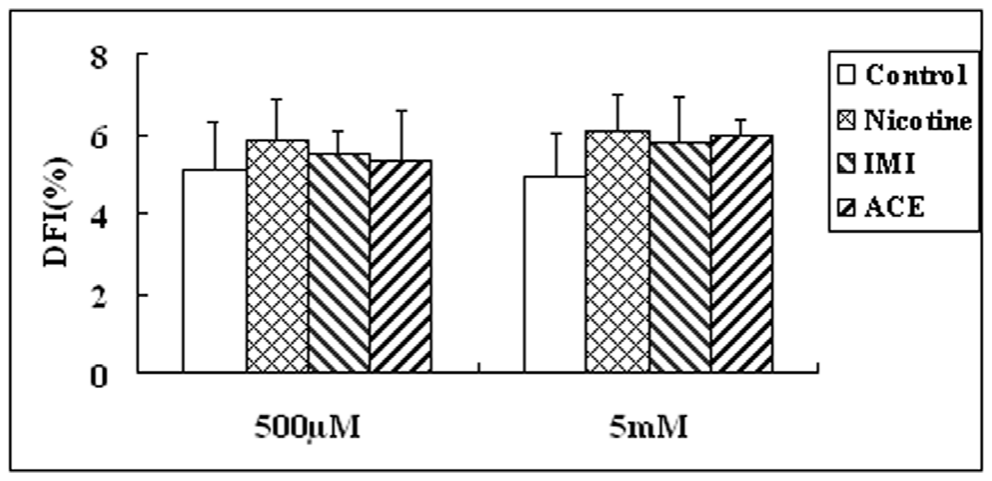
The SCD results of toxicants exposure upon sperm DNA integrity.

With CASA, we were able to obtain objective and quantitative descriptions of changes in sperm kinematic parameters in response to exogenous toxicant. Treated with 500 µM or 5 mM of chemicals for 30 minutes, motility of spermatozoa showed no obvious difference from that of control (data not shown). We then investigated whether toxicant-induced reproductive hazards was associated with sperm DNA lesion using SCD assay. All chemical-treated groups displayed a minor increase in average percentage of DNA fragmented spermatozoa compared with those of control groups without reaching a significant difference (*P*>0.05), as shown in Figure 1. With respect to the difference between 500 µM and 5 mM of each chemical, the response toward exogenous compounds at current differential concentration was not obvious, as similar effects on sperm motility and DFI were observed.

**Figure 2:**
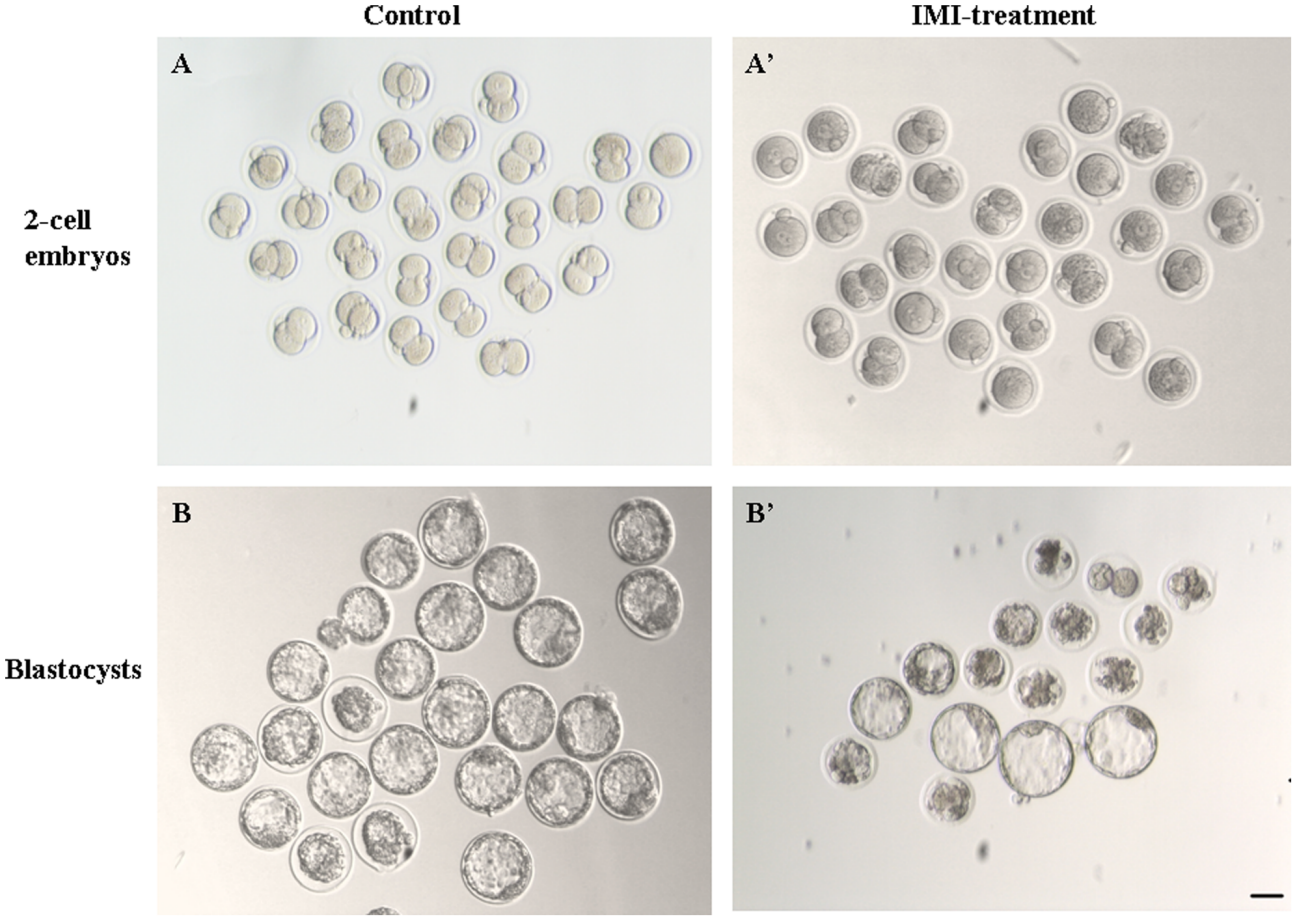
Partial embryos derived from IVF process with control or 5 mM IMI treated spermatozoa. 2-cell embryos derived from A) control and A’) IMI-treated spermatozoa, blastocytes derived from B) control and B’) IMI-treated spermatozoa. Scale bar = 50 µm.

When IVF was performed with the spermatozoa pretreated with chemicals adopted herein at the concentration of 500 µM or 5 mM, all fertilized oocytes survived without evident changes in cell morphology. Fertilized oocytes were judged normal by extrusion of second polar body and the presence of 2 pronuclei, which represents success of fertilization. Our preliminary data indicated that treatment with 500 µM of chemicals for 30 min did not induce any significant adverse effect on fertilization potential of the spermatozoa and subsequent embryo development (data not shown). However, during the culture process, embryos originated from spermatozoa pretreated with 5 mM of chemicals were more inclined to encounter failure of the first cleavage, wherein some of them exhibited various degrees of cellular fragmentation and asymmetry (Figure 2A’). Finally, when these embryos were cultured *in vitro* up to 96 h, with fragmentation, loss of cytoplasm or decrease in cytoplasmic clarity, part of them would arrest or degenerate during the developmental progression (Figure 2B’). As shown in Table 1, in the presence of 5 mM toxicant in HTF medium, all treated spermatozoa retained their potential to fertilize oocytes. However, in nicotine and IMI-exposed groups, rates of pronucleus formation (fertilization), the first cleavage and morula/blastocyst formation were remarkably decreased, compared to those of non-treated control (*P*<0.05). In ACE-exposed group, the first cleavage of zygote and blastocyst formation process were also impaired (91.2% and 58.5%, respectively), compared to those of the control group (98.5% and 74.6%, respectively), whereas the fertilization rate was slightly lower than that of control without reaching a statistical significance. Thus, ACE appeared to pose much weaker adverse effects on mouse spermatozoa, at least in terms of fertilization process *in vitro*. During embryo culture, embryo fragmentation, a process where portions of the embryo’s cells have broken off, had attracted our attention. It is preferable to have little or no fragmentation when evaluate a normal embryo, while nicotine and IMI exposure remarkably elevated the fragmented embryo percentage compared with control. Comparisons were also made among treated groups, it was observed that ACE exerted significantly moderate effect, whereas nicotine exposure showed most severe reproductive hazard.

**Table 1:** The effects of chemicals upon sperm fertilization capability in IVF procedure and subsequent embryonic development.

Category	No. of oocytes (replicates)	No. with pronuclei formation(%)^a^	No. of 2-cell embryos (%)^b^	No. of fragmented embryos (%)^b^	No. of morulae(%)^b^	No. of blastocysts(%)^b^
Control	283(5)	205(72.4)	202(98.5)	6(2.9)	165(80.5)	153(74.6)
nicotine-exposed	344(5)	186(54.1)*	155(83.3)*	31(16.7)*	93(50.0)*	78(41.9)*
IMI-exposed	301(5)	188(62.5)* ^, #^	160(85.1)*	16(8.5)* ^, #^	112(59.6)*	96(51.1)*
ACE-exposed	225(5)	159(70.7)^#,$^	145(91.2)* ^, #^	7(4.4)^#^	122(76.7)^#,$^	93(58.5)* ^,#^

a Based on total oocytes.

b Based on total pronuclear embryos.

* ^, #, $^ *P*<0.05, comparisons were made between control group and each treated group, nicotine group and two insecticide groups, and two insecticide groups, respectively.

### Inﬂuences of Chemical Exposure on Fertilization and Subsequent Embryonic Development *in vitro*

In consecutive exposure experiment with chemical exposure both in HTF and KSOM medium, the concentration of supplementation constricted to 500 µM in both of medium, which allowed the fertilized oocytes proceed to blastocysts without inducing excessive fragmented or dead embryos. It was showed that mixture of oocytes and spermatozoa in chemical-added HTF maintained normal fertilization capacity compared with control (ratio of oocytes with pronuclei formation, *P*>0.05), while the percentages of 2-cell embryo and morula/blastocyst formation decreased significantly (*P*<0.05). Among these three chemicals, ACE appeared most mild in the effect on the first cleavage (ratio of 2-cell embryo formation), with no significance found (Table 2).

**Table 2:** The effects of chemicals upon the consecutive exposure from fertilization to blastocyst formation.

Category	No. of oocytes (replicates)	No. of oocytes with pronuclei (%)^a^	No. of 2-cell embryos (%)^b^	No. of fragmented embryos (%)^b^	No. of morulae(%)^b^	No. of blastocysts (%)^b^
Control	132(3)	98(74.2)	94(95.9)	4(4.1)	79(80.6)	77(78.6)
Nicotine-exposed	138(3)	99(71.7)	82(82.8)*	10(10.1)	66(66.7)*	63(63.6)*
IMI-exposed	143(3)	102(71.3)	90(88.2)*	7(6.9)	68(66.7)*	67(65.7)*
ACE-exposed	142(3)	104(73.2)	95(91.3)	9(8.7)	70(67.3)*	68(65.4)*

a Based on total oocytes.

b Based on total pronuclear embryos.

* *P*<0.05, comparisons were made between control group and each treated group.

### Inﬂuences of Chemical Exposure on Preimplantation Embryonic Development *in vitro*

Considering that exposure of 1 mM chemicals would cause considerable embryo fragmentation or even death in our preliminary experiments (data not shown), we used 500 µM of concentration in culture medium. As shown in Table 3, exposure of these chemicals caused a moderate decline in the percentage of 2-cell embryo formation and a drastic impact on morula/blastocyst formation derived from normal zygotes, compared with control (*P*<0.05). When comparison was made among these three chemicals, exposure of IMI or ACE retarded embryonic development to a more moderate degree than nicotine, but no significant difference existed between IMI and ACE group. Taken together, adverse effects exerted on development of zygotes in the order of nicotine>IMI>ACE.

**Table 3:** The effects of chemicals upon the development of naturally fertilized zygotes.

Category	No. of oocytes withpronuclei (replicates)	No. of 2-cellembryos (%)^a^	No. of 4-cell embryos (%)^a^	No. of morulae(%)^a^	No. of blastocysts (%)^a^
Control zygotes	86(3)	82(95.3)	82(95.3)	81(94.2)	80(93.0)
Nicotine-exposed zygotes	125(3)	106(84.8)*	93(74.4)*	86(68.8)*	84(67.2)*
IMI-exposed zygotes	141(3)	134(95.0)^#^	123(87.2)* ^,#^	108(76.6)*	106(75.2)*
ACE-exposed zygotes	128(3)	120(93.8)^#^	113(88.3)^#^	105(82.0)* ^,#^	101(78.9)* ^,#^

a Based on total oocytes with pronuclei.

* ^, #^ *P*<0.05, comparisons were made between control group and each treated group, nicotine group and two insecticide groups, respectively.

Incubated with 500 µM of each chemical, development of naturally fertilized 2-cell embryos was also monitored (Table 4). Under these conditions, treatment with different chemicals did not show significant adverse effects on 2-cell embryos (*P*>0.05), with less extent of effects than those observed in zygotes. These results collectively revealed that the doses up to 500 µM of each chemical used in the study did not exert toxicity at the onset of 2-cell embryo, but fertilization or zygote as well as the subsequent developmental procedure with preceding chemical exposure.

**Table 4:** The effects of chemicals upon the development of 2-cell embryos.

Category	No. of 2-cell embryos (replicates)	No. of 4-cell embryos (%)^a^	No. of morulae (%)^a^	No. of blastocysts (%)^a^
Control 2-cell embryos	72(3)	71(98.6)	70(96.6)	70(97.2)
Nicotine-exposed 2-cell embryos	107(3)	104(97.2)	103(96.3)	100(93.5)
IMI-exposed 2-cell embryos	103(3)	102(99.0)	100(97.1)	98(95.1)
ACE-exposed 2-cell embryos	101(3)	100(99.0)	99(98.0)	95(94.1)

a Based on total 2-cell embryos.Haibin Wang.

## Discussion

Despite of lower affinity to mammalian nAChRs, neonicotinoid pesticides have been illustrated to impair mammalian reproduction by recent animal studies [5], [6], [7], [8], [9]. In the present study, we used a set of promising *in vitro* models of reproductive toxicology [19], and examined the direct effects of neonicotinoid pesticides, IMI and ACE, on spermatozoa, fertilization procedure and preimplantation embryo development.

Sperm quality, such as motility and DNA integrity, are important in male fertility and in the particular contribution to early embryonic development [20], which is also a sensitive and quick testing strategy for reproductive toxicology [21]. *In vitro* exposure of nicotine to human semen was reported to be able to cause human sperm DNA damage and motility decrease (1 mM for 20 min) [14], [22], [23]. However, we found that motility and DNA integrity were not significantly affected by a high exposure dosage (5 mM for 30 min) of chemicals, even with nicotine, which may result from difference of experimental objects, mouse spermatozoa *versus* human semen. When IVF process was introduced, subtle differences among the spermatozoa caused by pretreatment with different chemicals, nicotine, IMI or ACE, could still be detected through the procedures of fertilization and subsequent embryonic development (Table 1).

Procedure of IVF includes sperm-egg binding, zygote formation and the first cleavage to form 2-cell embryo. After being transferred into KSOM medium, 2-cell embryo could conduct multiple cleavages to form 4-cell embryo, morula then blastocyst successively *in vitro*. In order to determine the specific embryo developmental stages that chemicals could take effect, mixture of spermatozoa and eggs for fertilization, naturally fertilized zygotes and 2-cell embryos were separately prepared, and consecutive chemical exposure with a concentration of 500 µM was conducted until blastocyst formation. We observed that exposure of chemicals during fertilization could adversely affect 2-cell formation and subsequent embryo development (Table 2), normal zygotes with chemical exposure could impair subsequent 4-cell embryo formation and following procedure (Table 3), whereas there was no significant adverse effect on subsequent development when normal 2-cell embryos were treated with these chemicals (Table 4). Compared with the effects on fertilization procedure or zygotes, our results suggested that, 2-cell embryos were most resistant to exposure of 500 µM nicotine, IMI or ACE, which is consistent to the previous study of 2-cell embryos toxicity with nicotine [12].

Although it may cause human reproduction disorder, nicotine could show *in vitro* detriments only with a concentration much higher than the exposure level in an “average” smoker [12], [15], [24], suggesting that nicotine might adversely affect spermatozoa or embryos in an indirect way. Our study supported these previous reports, and implied that IMI and ACE may work with a similar mechanism to nicotine. Studies indicated that acute or chronic exposure of nicotine will cause oxidative stress in animal and human body [24], [25], [26], [27], which could do harm to reproductive organs. Several studies reported that oxidative stress caused by testicular tissue and lymphocyte in semen will impair sperm parameters [24], [27], suggesting that owing to lack of lymphocytes around, “swim-up” mouse spermatozoa in our study are more resist to the exposure of nicotine than human spermatozoa in semen. Human exposure to neonicotinoids is very limited (12.8–350 ng/ml in the urine of farm workers, with or without protection) [28], [29], our study indicated that, at exposure levels, IMI and ACE do not show adverse effects on mouse sperm functions and early embryo development *in vitro* (data not shown). However, recent animal studies showed that IMI and ACE could cause oxidative stress in body [5], [26], and even chronic exposure of IMI with a low concentration could result in oxidative stress in tissues [30], which warned that low level of neonicotinoid exposure in a long period may also exert impacts on reproduction of human, especially for professional populations.

Taken together, in a reproductive toxicity study, we herein integrated several *in vitro* tests reported in our and other previous studies [14], [19], [31], [32], which covered sperm quality, sperm penetration into oocytes, process of oocyte *in vitro* maturation, and preimplantation embryonic development. The results indicated that, at high levels, direct exposure of nicotine, IMI or ACE had harmful effects on sperm function and embryonic development, and stages mainly at fertilization, zygote formation, and first cleavage of zygote, with the extent in an order of nicotine>IMI>ACE. These preliminary but provocative results elucidated the reproductive toxicities of two neonicotinoid insecticides on mammals from a new prospective, which evaluated the direct effects of pesticides on gametes, fertilization and embryonic development. Meanwhile, our study suggested that application of an integrated testing strategy will help developers detect the reproductive toxicity and determine the targets in embryogenesis for a chemical substance sensitively and shortly.
